# Laparoscopic Sleeve Gastrectomy in a Patient With Situs Inversus Totalis and Kartagener Syndrome

**DOI:** 10.7759/cureus.17155

**Published:** 2021-08-13

**Authors:** Basem Almussallam, Saad M Alqahtani, Nael Abdo, Walid Maghraoui, Mohammad Fawaz, Asma Hachani, Sally D Soliman, Mohamad Elsayed, Saeed A Alsareii

**Affiliations:** 1 Department of Surgery, McMaster University, Hamilton, CAN; 2 Department of Surgery, College of Medicine, Majmaah University, Majmaah, SAU; 3 Department of General and Bariatric Surgery, National Medical Complex (NMC) Najran Hospital, Najran, SAU; 4 Department of Cardiology, National Medical Complex (NMC) Najran Hospital, Najran, SAU; 5 Department of Internal Medicine/Critical Care, National Medical Complex (NMC) Najran Hospital, Najran, SAU; 6 Department of Internal Medicine and Pulmonology, Alexandria Police Hospital, Alexandria, EGY; 7 Department of Anaesthesia, National Medical Complex (NMC) Najran Hospital, Najran, SAU; 8 Department of Surgery, College of Medicine, Najran University, Najran, SAU

**Keywords:** obesity, morbid obesity, situs, situs inversus, gastrectomy, sleeve gastrectomy, bariatric surgery

## Abstract

Laparoscopic sleeve gastrectomy (LSG) is a widely accepted and adopted procedure to achieves weight loss in morbid obesity. Situs inversus (SI) is when the body's visceral organs are not in the normal position with reversal of anatomical orientation. Patients with obesity and SI can be challenging to diagnose and manage. We present a case of a 23-year-old male who has SI totalis with Kartagener syndrome who underwent LSG to treat morbid obesity. Furthermore, we conducted a comprehensive review of the current medical literature. We conclude that LSG can be safely performed in SI. However, it is recommended to leave such cases to more experienced surgeons. In addition, it is advisable to consider few unconventional technical operative methods before surgery. Nevertheless, more data are needed to better study LSG in SI patients, which can be difficult given the rare nature of SI.

## Introduction

Situs inversus (SI) is a congenital condition characterized by the reversal of visceral organ orientation within the body [1-4]. Situs solitus is the term used when the visceral organs are in normal orientation [3,4]. SI can involve one or more organs. The genetics of SI is not fully understood but it is believed that most cases are due to sporadic mutation with evidence of other patterns of inheritance like autosomal recessive, autosomal dominant, and X-liked recessive [4-6]. SI has been described in mummies from historical anatomical museums [7]. Moreover, a similar condition of organ malposition was found in an ancient fetal Egyptian mummy who had dextrocardia [8]. There is an estimated one in 10,000 to one in 20,000 cases of SI [9].

Obesity is a growing health problem worldwide. According to the World Health Organization, more than half a billion adults are obese and around two billion adults are overweight in 2016 [10]. In 2017-2018, almost half of the adult population of the United States is considered obese with a prevalence of 42.4% [11]. Similar figures were reported in the younger age group (20 to 39 years old) with a 40.0% prevalence. Overall, there was no difference in prevalence between men and women. Obesity is linked to many health conditions that are potentially preventable. In the United States, the estimated annual medical cost related to obesity in 2008 was $147 billion with the medical cost of a patient with obesity being $1,429 higher compared to normal-weight patients [12]. Laparoscopic sleeve gastrectomy (LSG) is a widely accepted treatment modality in morbid obesity with substantial weight reduction specifically after the failure of other weight loss methods [13].

We present in this paper a case of a 23-year-old male who has SI totalis (SIT) with Kartagener syndrome who underwent an LSG to treat morbid obesity. We present the case and conduct a comprehensive review of the current medical literature with a focus on sleeve gastrectomy cases. We also discuss important clinical points that require attention in such cases.

## Case presentation

A 23-year-old male patient has morbid obesity with a weight of 146.3 kg and a height of 177 cm. The calculated body mass index (BMI) was 46.7 kg/m^2^. The patient had previously failed to lose weight using different dietary and lifestyle modification techniques over an approximately four years period. He provided a history of dextrocardia, bronchiectasis, and chronic sinusitis with no recent flare in the last three months. He denied any history of previous surgery, smoking or alcohol consumption. He elected to have LSG to help with weight reduction.

**Figure 1:**
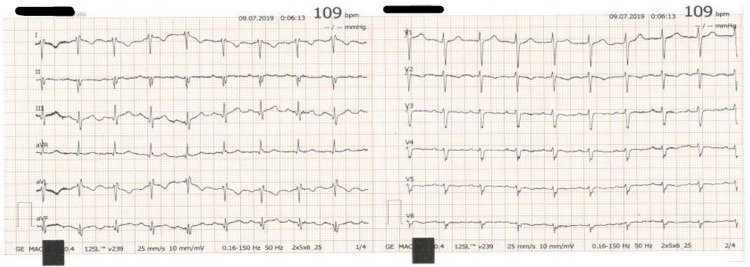
Electrocardiogram showing a right axis deviation.

**Figure 2:**
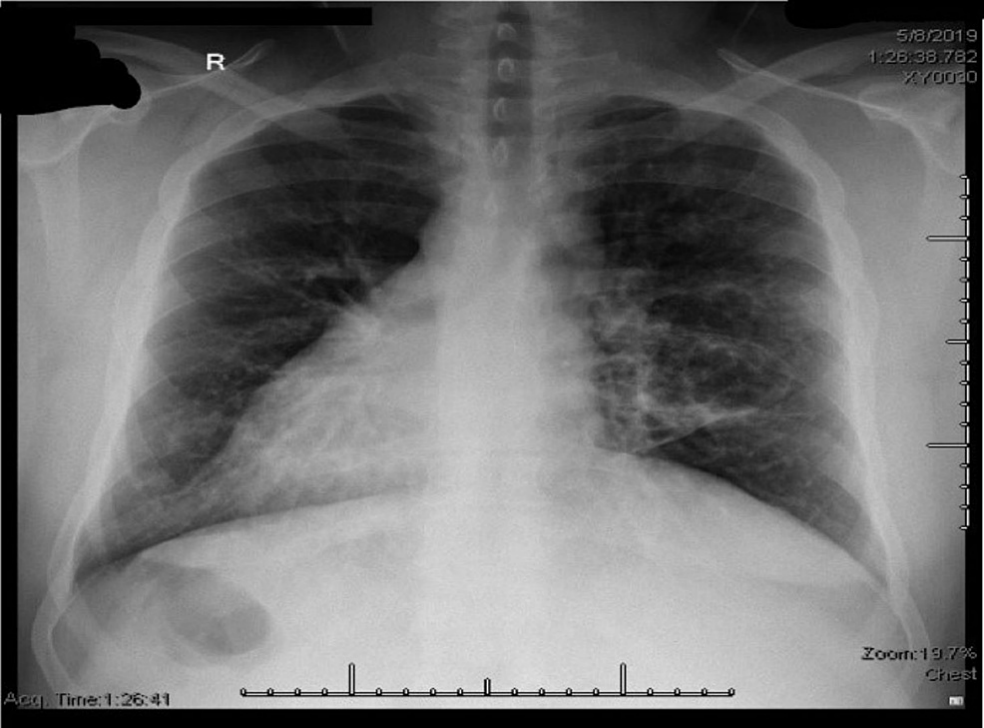
Chest x-ray showing dextrocardia.

**Figure 3:**
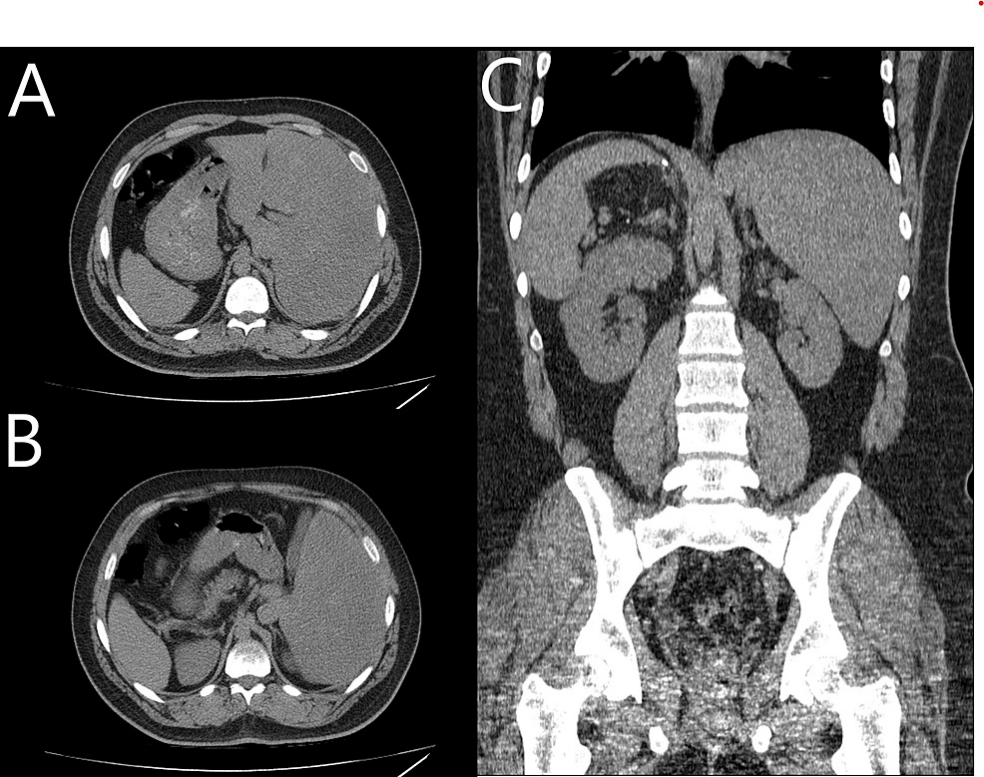
Computed tomography images of the abdomen with evidence of SIT. (A, B) Two cross-sectional views with the liver on the left upper quadrant and the stomach and spleen on the right. (C) A coronal view showing reverse orientation of abdominal organs.

**Figure 4:**
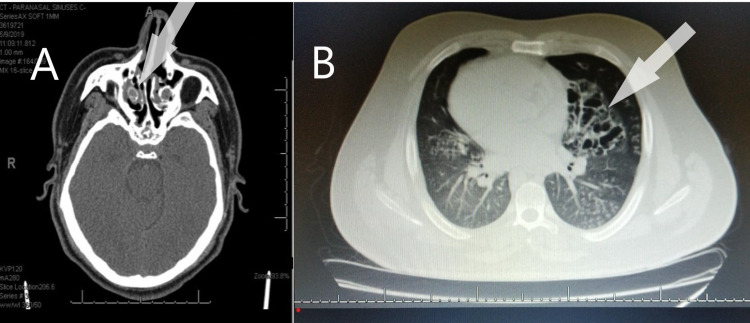
(A) A computed tomography scan of sinuses showing evidence of chronic sinusitis. (B) A computed tomography scan of the chest showing radiological features of bronchiectasis.

The patient was evaluated by a multidisciplinary bariatric surgery team, cardiology, pulmonology, and anesthesiology. He was cleared for the planned surgery. A full assessment was conducted preoperatively including physical exam, complete blood count, biochemical profile, thyroid function test, electrocardiogram, and pulmonary function test (PFT). Additionally, imaging studies including chest x-ray, transthoracic echocardiogram, ultrasound of abdomen, computed tomography (CT) scan of sinuses, chest, abdomen, and pelvis were performed. His blood workup was normal, the electrocardiogram revealed right axis deviation (Figure 1), and his radiological imaging showed evidence of SIT (Figures 2, 3A-3C). His echocardiogram was normal except for dextrocardia and he had mild restrictive airway disease on PFT. Evidence of chronic sinusitis was seen on his sinuses CT and his chest CT showed radiological features of SI and bronchiectasis (Figure 4).

 

 

 

**Figure 5:**
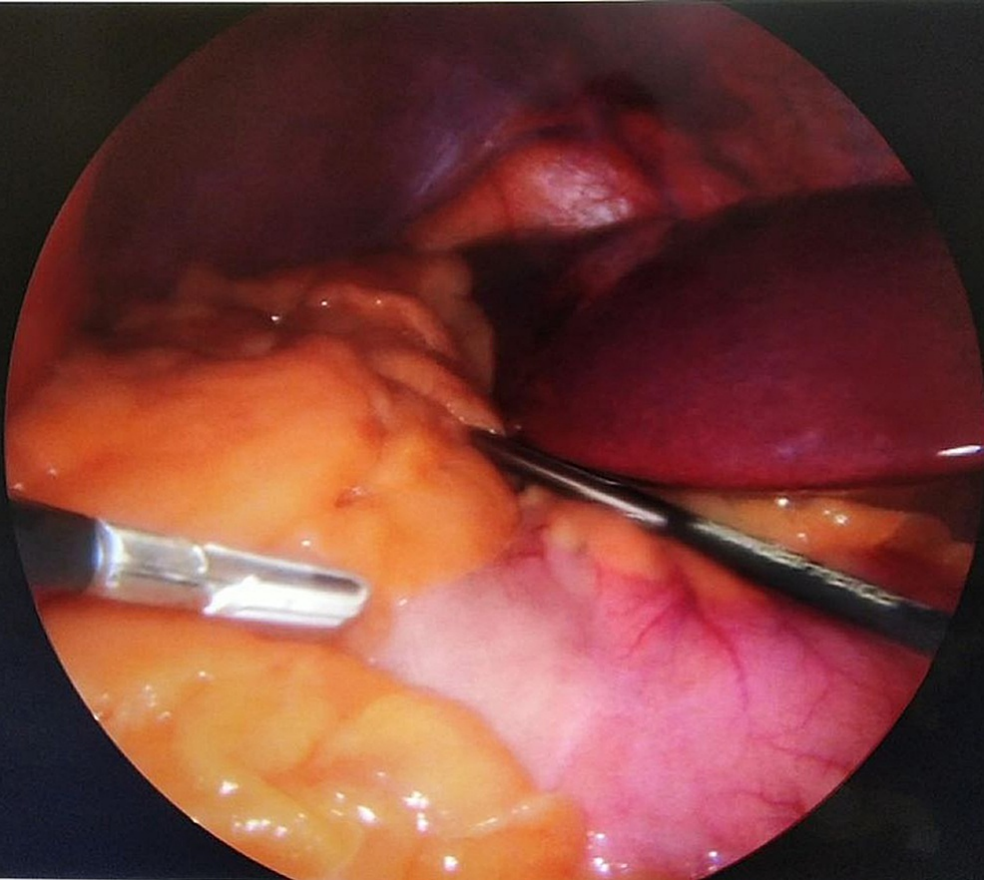
Intraoperative picture showing the liver on the left upper quadrant and the stomach on the right.

**Figure 6:**
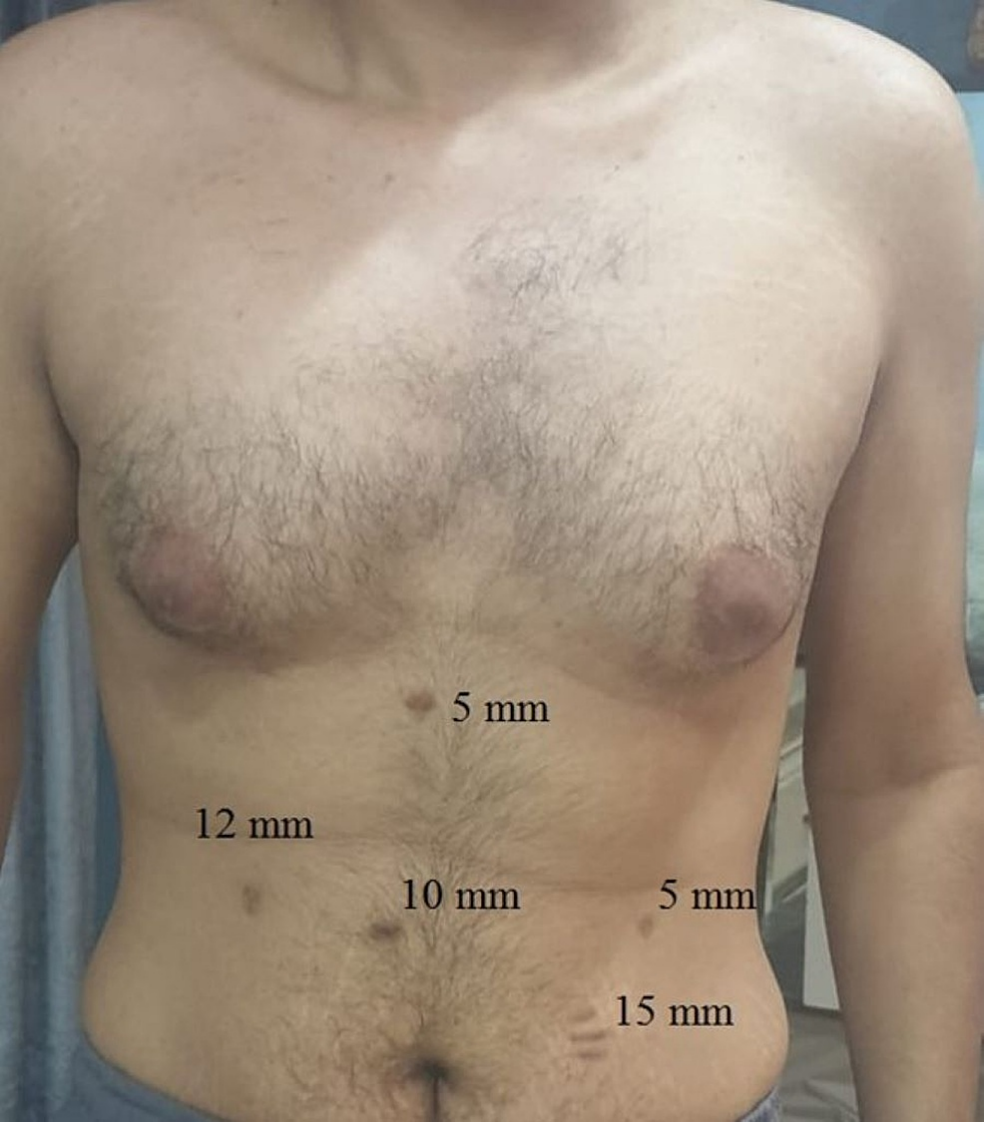
An illustration of the new placement sites of the laparoscopic ports.

He was admitted the night before surgery per pulmonology recommendation for preoperative monitoring. LSG was performed with the patient in the reverse Trendelenburg and French position (split-leg supine). The surgeon stood between the legs. The abdomen was accessed using veress needle technique inserted in the right upper quadrant at a mirror image of the palmer’s point after infiltration of local anesthesia. Pneumoperitoneum was achieved successfully and was maintained at 13 mmHg of intra-abdominal pressure. The veress needle was then removed and a 5-mm optical trocar was placed in the left lumbar region of the abdomen about 10-15 cm below the costal margin to avoid injuring the malpositioned liver. This was done under direct visualization using a 5 mm/zero-degree laparoscopic camera. Diagnostic laparoscopy revealed that all abdominal organs were in reverse orientation (Figure 5). The spleen was located in the right hypochondriac region. The gastric fundus and its greater curvature were found under the hepatic portion located to the right of the falciform ligament. Toward the left side, in the left hypochondriac region, the gallbladder was found under the hepatic portion located to the left of the falciform ligament. Other trocars were adapted to the new position (5 mm, 10 mm, 15 mm, and 12 mm) as shown in Figure 6. Using blunt tip LigaSure 5 mm-37 cm device, the dissection of omentum from great curvature was done starting 4 cm from pyloric sphincter, dissection of the short gastric vessel was done safely. GIA staplers (Covidien 60 mm one black cartilage and four purples) were used to resize the stomach using intragastric bougie of 36 Fr size. Suture line clipping was done to control oozing from the staple line using 5 mm clips. A Penrose drain was inserted at the end of the procedure. No leak test was done as part of our protocol for all LSG. Estimated blood loss at the end of the surgery was less than 100 mL. Removal of the stomach stump was done through the 15 mm port incision. Evacuation of pneumoperitoneum was done gradually and skin was closed with 4-0 vicryl subcticular stitches. Local anesthesia was infiltrated at the port incisions in the end. The total duration of the operation was 68 minutes, patient was extubated uneventfully and moved to the recovery room for monitoring.

 

No early surgical or anesthetic complications were reported during or after the operation. The patient started the bariatric clear liquid diet on postoperative day 1 and he was discharged home on postoperative day 2 after removing the drain. He was readmitted after two months postoperatively due to renal colic that resolved with non-operative management. He was on a full bariatric diet by then and he lost a total of 29.3 kg with his readmission weight measured at 117 kg. He continued to do well and showing good progress with his weight loss. His postoperative weight at one year was 88 Kg with a BMI of 28.1 kg/m^2^.

Literature review

A broad search of the published English-language medical literature utilizing Pubmed, Google Scholar, and Web of Science search engines for articles about LSG for weight loss in SI patients was performed. The following keywords were used: “obesity,” “morbid obesity,” “situs,” “situs inversus,” “gastrectomy,” “sleeve gastrectomy,” and “bariatric surgery.” The search included all articles from 1998 to May 2020. After a thorough screening, a total of 17 studies were found with 18 reported cases. Furthermore, we present a case on the same topic.

Key information extracted and summarized in Table 1 including our case. All cases had SIT except one case that had SI partials [14] and another that had situs ambiguous [15]. Four cases had Kartagener syndrome [16-18]. One case had a planned additional procedure [19] and another had an unplanned additional procedure [15]. Multiple port sites were used except for one case that utilized a single incision LSG technique [16]. Additional ports were needed in two cases [19,20] with four cases missing that information [14,21,22]. French position used nine times [16,19,20,23-27]. The primary surgeon stood between the legs in eight cases [16,20,23-27], to the left of the patient in six cases [14,15,17,21,28] and to the right in three cases [18,19,22]. The surgeon position was missing in two cases [29,30]. The mean age of cases was ~39 years (range 19-59). The patients were predominantly females (15 females and four males). The mean BMI was 45.9 kg/m^2^ (range 36-76). The mean operative time was ~75 minutes (range 28-202) but four authors did not report this information [15,18,20,29] and Watanabe et al. had a very long operative time due to the additional procedure [19]. The mean postoperative hospital length of stay was ~3 days (range 1-7) with three articles not reporting the length of stay [16,21,29]. Preoperative workup looked similar between groups with some variations. Most of the cases had obesity-related comorbidities. Five cases had prior surgeries [19,25,27-29] with a single article that did not provide surgical history [17]. Only three cases had a perioperative complication [15,18,29]. Intraoperative spleen infarction was reported by Shaheen et al. of four out of six spleens in a patient with situs ambiguous and polysplenia [15]. This was recognized during the time of LSG and was successfully resected. Deutsch et al. described a postoperative suture/staple line leak that was managed with endoscopic poly flex stenting [29]. Mosquera et al. reported upper gastrointestinal bleeding after being discharged from the hospital on postoperative day 5 [18]. This resolved spontaneously with clinical monitoring without the need for blood product transfusion. All of the cases achieved variable but significant postoperative weight loss. Data on weight loss were lacking in five cases [16,21,26,30].

**Table 1:** Summary of literature review of cases of LSG in SI patients. NA=Not Available, M=Male, F=Female, SIT=Situs Inversus Totalis, SIP=Situs Inversus Partials, SA=Situs Ambiguous, BMI=Body Mass Index, HTN=Hypertension, DM=Diabetes Miletus, OSA=Obstructive Sleep Apnea, DLD=Dyslipidemia, NAFL=Non-Alcoholic Fatty Liver, EKG=Electrocardiogram, CXR=Chest X-ray, EGD=Esophagogastroduodenoscopy, Abd US= Abdominal Ultrasound, Abd CT=Abdominal Computed Tomography, Chest CT= Chest Computed Tomography, CT=Computed tomography, UGI=Upper Gastrointestinal Series Study, Sinuses CT= Sinuses Computed tomography, PFT=Pulmonary Function Test, SILSG=Single Incision Laparoscopic Sleeve Gastrectomy, LOS=Hospital Length of Stay Postoperatively, GI= Gastrointestinal, EWL=Excess Weight Loss.

Author cases		Age/Gender	Situs Type	Kartagener Syndrome	BMI (Kg/m2)	Co-morbidities	Previous Surgeries	Preoperative Workup	Surgical Position/ Surgeon Position	Operative Time (minutes)	Unique/ Additional procedure	Additional ports	LOS (days)	Reported Complications	Weight loss
Catheline et al. (2006) [20]		19/M	SIT	No	76	Asthma, Enuresis, Arthritis	No	EKG, CXR, Echocardiogram, EGD, Abd US	French/ between legs	NA	No	Yes	7	No	EWL 39% in 9 months
Borude et al. (2012) [14]		32/F	SIP	No	41.4	None	No	Barium follow through, Abd CT, Chest CT	Supine/ Left side	90	No	NA	4	No	New BMI 28 in 1-2 years
Deutsch et al. (2012) [29]		39/F	SIT	No	42	HTN, DM, OSA, Hypothyroidism	laparoscopic converted to Open gastric banding	Abd CT	Supine/ NA	NA	No	No	NA	Suture/ staple line leak	EWL 60% in 2 years
Stier et al. (2014) [23]		51/F	SIT	No	54.2	HTN, DM	No	EKG, CXR, EGD, Abd US	French/ Between legs	61	No	No	5	No	New BMI 40.3 in 2 years
Genser et al. (2015) [16]		52/F	SIT	Yes	49	OSA, liver steatosis, H. Pylori Gastritis	No	EKG, CXR, Abd CT	French/ Between legs	45	SILSG	No	NA	No	NA
Yazar et al. (2016) [24]		21/F	SIT	No	41.8	No	No	EKG, CXR, EGD	French/ Between legs	78	No	No	6	No	New BMI 29.8 in 8 months
Watanabe et al. (2016) [19]		46/F	SIT	No	40.3	HTN, DLD, DM, OSA, NAFL, Mental disorders, H. Pylori Gastritis	Appendectomy, Hysterectomy	EKG, EGD, CT	French/ Right side	202	DJB	Yes	3	No	New BMI 24.5 in 1 year
Shaheen et al. (2016) [15]		38/F	SA	No	55.8	Polysplenia	No	“Cardiac workup”, CXR, EGD, Abd US, Abd CT	Supine/ Left side	NA	Resection of four infarcted mini-spleens	No	1	intraoperative infarction of multiple mini-spleens (2 large spleen remained intact)	EWL 49.9% in 6 months
Aziret et al. (2016) [25]		54/F	SIT	No	48	DM, Arthritis	Open cholecystectomy	CXR, Abd CT	French/ Between legs	105	No	No	5	No	EWL 51.7% in 4 months
Salerno et al. (2017) [26]		41/M	SIT	No	46.4	No	No	“Similar to standard anatomy”	French/ Between legs	45	No	No	2	No	NA
Taha et al. (2018) [21]	Case 1	33/F	SIT	No	42.7	No	No	EKG, CXR, Barium swallow	Supine/ Left side	50	No	NA	2	No	NA
	Case 2	41/F	SIT	No	41.7	Asthma	No	EKG, CXR, Barium swallow	Supine/ Left side	75	No	NA	NA	No	NA
Froylich et al. (2018) [30]		47/F	SIT	No	51	Asthma, HTN, DM	No	CXR, UGI	Semi-lithotomy/ NA	62	No	No	2	No	NA
Villalvazo et al. (2018) [28]		59/F	SIT	No	38	Pre-diabetic, OSA, Degenerative joint disease, liver steatosis	Cesarean section, evacuation of ectopic pregnancy	Abd XR, Echocardiogram, EGD, UGI, Abd US, Abd CT, PFT	Supine/ Left side	108	No	No	3	No	New BMI 29 in 3 months
Burvill et al. (2019) [17]		25/F	SIT	Yes	40	Lactose intolerance	No	NA	NA/ Left side	35	No	No	2	No	EWL 125% in 1 year
Ali et al. (2019) [22]		48/F	SIT	No	41	No	NA	EGD, Abd CT, Chest CT	Supine/ Right side	75	No	NA	2	No	NA
Mosquera et al. (2020) [18]		47/M	SIT	Yes	40.9	HTN, DM, OSA, Arthritis, Chronic Sinusitis, nasal polyps	No	CXR, Echocardiogram, EGD, Nasosinuscopy	NA/ Right side	NA	No	No	2	Upper GI bleeding in postop day 5 (managed non operative)	New BMI 29.7 in 1 month
Bawahab et al. (2020) [27]		30/F	SIT	No	36	DM	Cesarean section	EKG, CXR, Echocardiogram, Barium swallow, Abd US	French/ Between legs	28	No	No	1	No	New BMI 24 in 1 year
Current case (2020)		23/M	SIT	Yes	46.7	Chronic sinusitis, Bronchiectasis	No	EKG, CXR, Echocardiogram, Abd US, Abd CT, Chest CT, Sinuses CT, PFT	French/ Between legs	68	No	No	2	No	EWL 41.1% in 2 months

## Discussion

SI cases can go undiagnosed in their adulthood especially if they have no associated anomalies or heart defects. However, SI can be diagnosed incidentally while performing routine imaging or electrocardiogram for an unrelated problem. Patients presenting to the surgery clinic may fail to mention that they have SI either because they think it is not relevant or because they are not aware of it. Surgeons need to pay attention and take a thorough history and ask the patient if they were told before that they have an unusual condition. Also, family history may help but not always as SI can happen due to sporadic genetic mutations. If the localization of the symptoms does not make sense then this should trigger a high clinical suspicion of SI. A careful physical exam can reveal the congenital condition especially if the patient has dextrocardia with heart sounds more prominent in the right chest.

Imaging such as ultrasound or plain chest x-ray will easily discover the condition in case the diagnosis was missed in the physical exam. Nevertheless, SI can present in different degrees and make it more difficult to diagnose even radiologically. It can be complete which is called SIT. SIT involves all the body organs in a mirror image orientation of the normal body. In SIT type the diagnosis should not be difficult. In contrast, it can be partials, ambiguous, or hetrotaxy based on the organ involved, a pattern of lateralization, the body cavity involved, and absence of body viscera or organs in some cases [1,3,4]. Hence, good attention and more time spent on these patients by the clinicians and the radiologist is essential.

It is particularly important to be able to identify SI patients not only for diagnostic purposes but also for the planning of clinical care and surgical approach. These conditions can be associated with other diseases and defects that can affect the patient health. Congenital heart diseases are not uncommon especially if dextrocardia is present. This can include atrial or ventricular septal defects, an anomaly of great vessels, coronary sinus absence, double-inlet/outlet ventricle, and pulmonary valve and veins defects [1,3,31]. Therefore, echocardiograms and vigilant cardiac assessments are essential prior to any intervention. In addition, like our case, SI can be associated with other conditions such as Kartagener syndrome where the motility of the cilia in the body is affected including the lungs. In this case, the risk of pulmonary complications may be increased [32].

Surgical planning should start with early referral to an anesthesia clinic for preoperative assessment. SI can raise a couple of anesthetic-related concerns and issues [24,33]. First, a low threshold to refer to Cardiology and proper cardiac assessment is mandatory as congenital heart defects can be present. Second, other possible associated syndromes should be excluded such as Kartagener syndrome or airway anomaly that can affect intubation, ventilation or increase pulmonary-related complications [32]. The anesthetist should be prepared to deal with increased respiratory secretions, bronchospasm, respiratory infections, and prolonged intubation. Suctioning should be more frequent and medication such as hydrocortisone and bronchodilators to decrease the chance of bronchospasm should be readily available. Third, the position to place the electrocardiogram leads for monitoring or cardiac defibrillator pads in case of cardiac arrest should not be in the usual fashion in case of dextrocardia. It should be placed in the right chest wall in a mirror image of the regular placement position because of the heart malposition in the right chest cavity. Finally, careful attention should be made to the patient's full recovery from anesthesia and return of spontaneous respiration prior to extubation as there have been reports of cases of prolonged postoperative apnea in SI [33].

Surgeons need to pay attention to several points prior to taking a SI patient for surgery. LSG has been proven to be safe in SI [14,16,25-30,17-24]. From the 19 reported SI patients who had LSG, only three cases had complications [15,18,29]. Those complications were easily identified and successfully managed. In Deutsch et al. case, the leak was contributed to difficult dissection secondary to adhesion from previous failed laparoscopic converted to open gastric banding [29]. Management included endoscopic poly flex stent placement and clinical observation. Interestingly, a review of bariatric surgery in SI revealed another complication related to gastric banding with band erosion [18]. The intraoperative four mini-spleens infarcts that developed in the case reported by Shaheen et al. might be related to the different anatomy and pathophysiology of situs ambiguous [15]. Those infarcted mini-spleens may have their blood supply solely coming from the arteries that are dissected during LSG. Two of those four spleens were wandering after surgical dissection and they twisted around themselves. Regardless, the patient had a total of six spleens with two larger remaining healthy spleens that most likely are receiving their blood supply from the splenic artery. The third case that had complications developed self-limiting upper gastrointestinal bleeding on a postoperative day 5 after he was discharged from the hospital [18].

In SI, the reversal of the body viscera orientation can be challenging to any surgeon who is planning LSG. Identification of anatomic landmarks and surgical dissection can be challenging. Port placement will be modified and most of the dissection will be from the right side of the body because of the stomach position. In contrast, Salerno et al. presented a successful LSG in SI with no alteration of port placement or surgical technique [26]. Additional port placement can be utilized for additional traction or easy access as described in two cases of LSG in SI [19,20]. Genser et al. have suggested trans-umbilical single incision LSG as a method to overcome anatomical variation of SI cases [16]. In contrast, vertical endoscopic gastroplasty in equipped centers has been proposed as an alternative procedure for weight loss in SI [34].

One major challenge is related to cognitive adaptation of the reversed anatomy during surgery with a suggested solution including a thorough review of preoperative radiological imaging [28] and a set of remainders and cross-checks by the surgical team [17]. Procedure time can be a challenge especially when adhesions from previous surgery are present [18,23-25] with surgeons understandably cautious given the alteration of the patient anatomy. The technical aspects of the surgery seem to be the hardest component with the possibility of left-handed surgeons having some advantage in SI cases as complaints were reported about the need to use the opposite hand [14,18-20,22,28].

Different surgical positions can be utilized in SI patients undergoing LSG. Nonetheless, the French position used in our case seems to be the most preferred. The French position is believed to provide easy access to the umbilical area and can possibly minimize the effect of reverse anatomy [23,24,27]. More importantly, these cases should be left to the more experienced surgeons with the availability of support if needed as these cases may require additional assistance, identification of anatomical variance, conversion to open surgery, repair of unexpected intraoperative injuries, or managing postoperative complications [14,17,18,23-27,29,30]. Robotic surgery has been suggested as a way to deal with the reverse anatomy in SI [18] given the enhanced dexterity, better ergonomic, and superior 3-D visualization. This needs to be further explored as to date, there is only one reported case of robotic-assisted Roux-en-Y gastric bypass [35], and no robotic cases reported for sleeve gastrectomy for weight loss in SI. Finally, more data is needed to better study LSG in SI patients, which can be difficult given the rare nature of SI.

## Conclusions

SI patients with morbid obesity can be safely managed and treated. Attention is needed to clinical hints in history, examination, and imaging to identify SI and avoid misdiagnosis. Management can be challenging to the anesthetist and the surgeon especially if associated conditions are present, such as Kartagener syndrome or congenital cardiac anomaly. Careful planning and cooperation between the surgical teams and different specialties are key. LSG can be safely performed in SI. However, it is recommended to leave such cases to more experienced surgeons. In addition, it is advisable to consider few unconventional technical operative methods before surgery. Finally, more data are needed to better study LSG in SI patients, which can be difficult given the rare nature of SI.
